# Zero-Shot Traffic Sign Recognition Based on Midlevel Feature Matching

**DOI:** 10.3390/s23239607

**Published:** 2023-12-04

**Authors:** Yaozong Gan, Guang Li, Ren Togo, Keisuke Maeda, Takahiro Ogawa, Miki Haseyama

**Affiliations:** 1 Graduate School of Information Science and Technology, Hokkaido University, N-14, W-9, Kita-ku, Sapporo 060-0814, Japan; gan@lmd.ist.hokudai.ac.jp; 2 Education and Research Center for Mathematical and Data Science, Hokkaido University, N-12, W-7, Kita-Ku, Sapporo 060-0812, Japan; guang@lmd.ist.hokudai.ac.jp; 3 Faculty of Information Science and Technology, Hokkaido University, N-14, W-9, Kita-ku, Sapporo 060-0814, Japan; togo@lmd.ist.hokudai.ac.jp (R.T.); maeda@lmd.ist.hokudai.ac.jp (K.M.); ogawa@lmd.ist.hokudai.ac.jp (T.O.)

**Keywords:** zero-shot traffic sign recognition, traffic sign matching, midlevel feature

## Abstract

Traffic sign recognition is a complex and challenging yet popular problem that can assist drivers on the road and reduce traffic accidents. Most existing methods for traffic sign recognition use convolutional neural networks (CNNs) and can achieve high recognition accuracy. However, these methods first require a large number of carefully crafted traffic sign datasets for the training process. Moreover, since traffic signs differ in each country and there is a variety of traffic signs, these methods need to be fine-tuned when recognizing new traffic sign categories. To address these issues, we propose a traffic sign matching method for zero-shot recognition. Our proposed method can perform traffic sign recognition without training data by directly matching the similarity of target and template traffic sign images. Our method uses the midlevel features of CNNs to obtain robust feature representations of traffic signs without additional training or fine-tuning. We discovered that midlevel features improve the accuracy of zero-shot traffic sign recognition. The proposed method achieves promising recognition results on the German Traffic Sign Recognition Benchmark open dataset and a real-world dataset taken from Sapporo City, Japan.

## 1. Introduction

With the increasing number of vehicles on the road, ensuring traffic safety has become essential in our daily lives [1]. According to the World Health Organization, road traffic accidents cause approximately 1.3 million deaths and 20–50 million nonfatal injuries each year (https://www.who.int/health-topics/road-safety, accessed on 16 October 2023). Reducing the occurrence of traffic accidents is crucial not only to protect people’s lives but also to maintain social stability. As an essential component of road traffic, traffic signs can provide drivers with important road information. However, traffic sign recognition is a complex task that is often affected by weather and road conditions [2] and is usually applied in Driver Assistance Systems (DASs) (for the abbreviations in this paper, refer to Appendix A Table A1) [3]. Based on traffic sign information, DASs can determine the driving environment and alert drivers of any mismatches that occur, which can help construct active vehicle safety systems in critical conditions. Moreover, traffic sign information can also help GPS providers with updating their geodatabases. Therefore, it is worth exploring efficient methods for accurate traffic sign recognition.

Traffic sign recognition has attracted widespread interest, and many related methods have been proposed [4,5,6] to address it. Before the era of deep learning, several studies used genetic algorithms [7] or shallow neural networks to perform traffic sign recognition [8,9]. Other methods obtain the features of traffic signs using a feature extraction algorithm like scale-invariant feature transform (SIFT) [10] and the histogram of oriented gradient (HOG) [11]. Although these conventional methods can recognize traffic signs to some extent, their computational efficiency and accuracy are still insufficient [12,13]. With the development of deep learning, research on traffic sign recognition has been focused on two aspects: traffic sign detection (TSD) and traffic sign classification (TSC) [14,15,16]. TSD uses object detection networks to detect traffic signs from road images, and TSC classifies the traffic signs [17]. Some TSC methods based on convolutional neural networks (CNNs) have been proposed [18,19,20] and achieved high classification accuracy. These methods rely on a large amount of carefully crafted data for training. However, as it costs time to capture many images containing traffic signs, there may be situations where there is not enough training data. Furthermore, traffic signs differ from country to country, and these methods need to be fine-tuned when recognizing traffic signs in different countries. Therefore, solving the traffic sign recognition problem within these settings is necessary.

**Figure 1:**
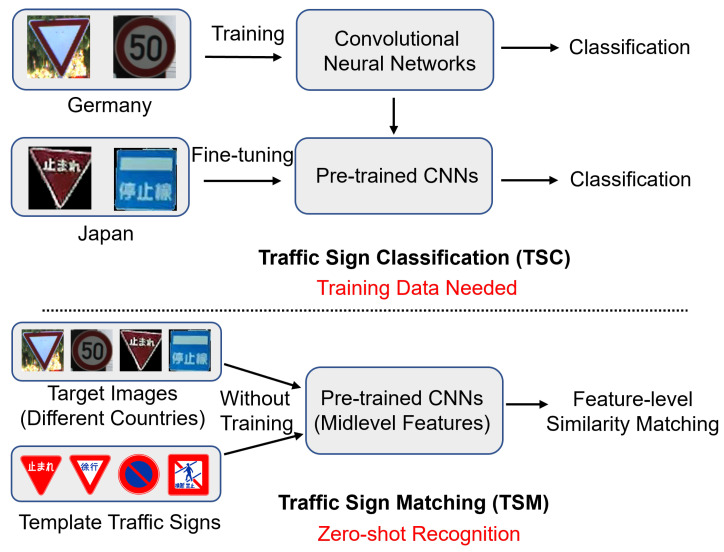
Concept of the proposed zero-shot traffic sign recognition method. This method can perform traffic sign recognition for different countries without collecting training data. Common traffic sign templates for each country can be used.

As shown in Figure 1, due to the lack of adequate training data, recognizing traffic signs from various countries through simple classification is challenging. However, unlike TSC, traffic sign matching (TSM) performs traffic sign recognition by matching the similarity of target and template traffic sign images without training data. The national standard traffic sign template database provides an easy source of template traffic signs for this purpose. This approach enables the recognition of traffic sign images from different countries. Although a TSM method exists [3], it is difficult to achieve high-accuracy TSM using the handcrafted features of SIFT. Midlevel features have proven effective in representing image features for other tasks [21,22], but no such method exists for traffic sign recognition to the best of our knowledge.

To solve the aforementioned problems, we propose a novel method based on midlevel feature matching to achieve accurate zero-shot traffic sign recognition. We found that compared with other layers, the midlevel features of CNNs not only obtain semantic information but also retain the shape information of traffic signs. We performed the extraction of midlevel features using different CNN structures, and the proposed method can obtain the robust feature representation of traffic signs without modifying any network structure. Moreover, since no training and fine-tuning is required, the proposed method based on midlevel features can be easily applied to traffic sign recognition in different countries.

Our contributions are listed as follows:

- We propose a novel TSM method for zero-shot recognition, which can achieve high-accuracy traffic sign recognition without additional training data.
- We introduce midlevel feature matching for the first time and perform the extraction of midlevel features on several CNN structures.
- We realize promising traffic sign recognition results on the German Traffic Sign Recognition Benchmark open dataset and a real-world dataset taken from Sapporo City, Japan.

## 2. Related Works

Traffic sign recognition has been extensively studied, and various approaches have been proposed to address this task. In this section, we provide an overview of related works in the areas of traditional methods and deep-learning-based approaches, highlighting their contributions and limitations. In Section 2.1, Section 2.2, and Section 2.3, we, respectively, introduce traditional traffic sign recognition methods, deep-learning-based traffic sign recognition methods, and TSM.

### 2.1. Traditional Traffic Sign Recognition Methods

Traditional methods for traffic sign recognition often relied on handcrafted features and machine learning algorithms. These approaches employed techniques such as template matching, edge detection, and feature extraction to recognize traffic signs. For instance, SIFT features were widely used to capture distinctive key points and descriptors of traffic signs [10]. Other methods, like HOG, utilized gradient-based image features to represent traffic signs [11]. These handcrafted features were then fed into classifiers such as support vector machines or decision trees for recognition [23,24,25,26].

Traditional traffic sign recognition methods have several characteristics and limitations. First, they heavily rely on manually designed features that are sensitive to variations in lighting conditions, occlusions, and complex backgrounds [27]. Second, these methods often struggle to adapt to diverse traffic sign datasets and real-world scenarios, as the handcrafted features may not generalize well [28]. Despite these limitations, traditional methods served as the foundation for early traffic sign recognition research and demonstrated reasonable performance under controlled conditions [29,30,31,32].

### 2.2. Deep-Learning-Based Traffic Sign Recognition Methods

The emergence of deep learning has inspired traffic sign recognition and has led to significant advancements in accuracy and robustness. Deep-learning-based approaches leverage CNNs to automatically learn hierarchical representations from raw image data, capturing both low-level visual features and high-level semantic information. Various CNN architectures have been explored for traffic sign recognition, including LeNet [33], AlexNet [34], VGGNet [35], and ResNet [36]. These architectures help to perform feature extraction and have contributed to the remarkable success of deep learning in traffic sign recognition [37,38,39,40,41,42,43]. To address the challenge of limited training data in traffic sign recognition, data augmentation techniques have been employed to expand the training set artificially. Common augmentation techniques include random rotations, translations, and scaling, as well as adding noise or distortions to the images. These techniques enhance the generalization ability of deep learning models and mitigate the risk of overfitting [44,45].

Moreover, recent studies have investigated the fusion of multimodal information for traffic sign recognition. For instance, combining visual information with temporal information from video sequences can significantly improve detection and classification performance, especially in dynamic traffic environments [46,47,48,49].

Despite remarkable results, deep-learning-based approaches still face challenges. They require substantial amounts of annotated data for training, which can be expensive and time-consuming to acquire. In addition, the lack of interpretability in model decisions raises concerns in safety-critical applications. Overcoming these challenges remains an essential task of research in traffic sign recognition [48].

### 2.3. Traffic Sign Matching Methods

TSM approaches offer an alternative perspective to traffic sign recognition by focusing on the similarity matching between target and template traffic sign images. Instead of relying on labeled training data, TSM methods utilize template traffic signs obtained from standardized traffic sign databases as references [3].

Early TSM methods often relied on handcrafted features like SIFT or speeded-up robust features (SURF) to extract local descriptors and perform similarity matching [3,50]. Ren et al. [3] introduced the conversion of urban road images from the RGB to HSV color space. SIFT and SURF features are then employed to compare the candidate traffic signs with template signs provided in the database. Peker et al. [51] presented a high-performance and robust system that involves RGB and depth images, along with template matching to perform TSM. However, these methods faced challenges in achieving high accuracy, especially when dealing with variations in scale, viewpoint, and lighting conditions.

Midlevel feature matching has demonstrated effectiveness in other computer vision tasks, such as scene recognition and object retrieval [21,22]. Aslam et al. introduced a midlevel feature-based method for representing images in classification problems and achieved higher classification accuracy. Furthermore, Gordo et al. [52] introduced the method of local midlevel features based on SIFT, which can construct fixed-length features for image representation. Lim et al. [53] introduced sketch tokens for learning-based midlevel representation in contour and object detection. Sketch tokens utilize supervised midlevel information in the form of hand-drawn contour sketches from images. Liu et al. [54] proposed a novel midlevel feature learning method for skin lesion classification, which acquires midlevel feature representations by learning the relationships between different image samples based on distance metrics. Zhong et al. [55] introduced a method based on midlevel features to predict facial attributes from faces in the wild, which achieved superior prediction accuracy compared with high-level features. The application of midlevel features to traffic sign recognition is the novel contribution of our work. The midlevel features can extract robust and discriminative representations of traffic signs. Unlike low-level features, midlevel features capture more abstract information, enabling the model to discern intricate patterns crucial for traffic sign recognition. Additionally, midlevel features often exhibit better generalization across diverse conditions compared with high-level features, making them well-suited for real-world applications with varying environmental factors. Using the feature representation capabilities and robustness of midlevel features, our method aims to achieve precise recognition of traffic signs from different countries without the necessity for fine-tuning or retraining.

## 3. TSM Method Using Midlevel Features

**Figure 2:**
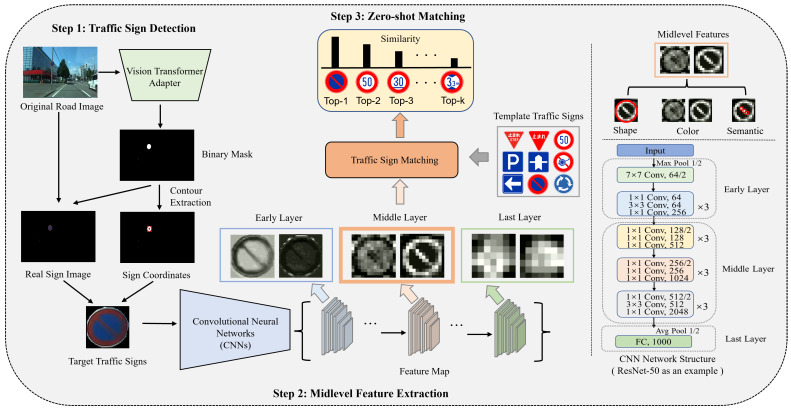
Overview of the proposed traffic sign recognition method. We first extract the target traffic signs from the road images based on ViT-Adapter. We then use the midlevel features of CNNs to perform TSM.

As shown in Figure 2, this section provides an insightful overview of the envisioned traffic sign recognition method. The method is designed to not only extract target traffic signs from original road images but also derive their feature representations through the extraction of midlevel features from CNNs, facilitating TSM. The proposed method unfolds through a coherent sequence of three fundamental steps: TSD, midlevel feature extraction, and zero-shot matching, each meticulously detailed in subsequent Section 3.1, Section 3.2, and Section 3.3.

### 3.1. Traffic Sign Detection

The TSD process represents the initial phase of our method and plays a vital role in precisely localizing traffic signs within complex urban road images. In this section, we delve deeper into the techniques employed for TSD. We employ a Vision Transformer Adapter (ViT-Adapter) [56], an innovative approach inspired by recent advances in vision transformers [57]. The ViT-Adapter is meticulously tailored for object identification within images and thus inspires us to extend its capabilities to the specific task of TSD. This adaptation involves introducing inductive deviations, which help recognize and categorize traffic signs within road scenes effectively. The initial step involves inputting the original road images into the ViT-Adapter. The ViT-Adapter performs well in producing segmented images that represent various object categories in meticulous detail. In our context, these object categories correspond to different types of traffic signs. Within these segmented images, various traffic signs within urban road scenes are meticulously color-coded. Each distinct color corresponds to a specific object category, facilitating their recognition and differentiation. To further refine the ViT-Adapter’s outputs for the purpose of distinguishing traffic signs, we convert these color-coded images into binary masks. This transformation simplifies subsequent processing steps and provides a clear delineation of traffic sign areas. The binary encoding effectively separates traffic signs from the background and other objects within the scene, significantly enhancing the detection and recognition of traffic signs.

After obtaining the binary masks, we employ contour detection algorithms [58] to precisely delineate the boundaries of the traffic signs. Suzuki et al. [58] introduced a topological structural analysis of digitized binary images using a border-following technique. Given a binary image *N* represented as a grid of pixels, where each pixel is either foreground (representing the traffic sign) or background (representing the surroundings), the contour detection algorithm identifies the connected components of the foreground pixels. Subsequently, it traces the borders of these components, effectively outlining the shape of the detected traffic sign $I_{d}$. The calculation process can be expressed as follows:

(1) $$I_{d}=\left\{\left(i,j\right)|N_{ij}=1\right\},$$

where $N_{ij}$ represents the foreground pixel of the traffic sign.

The contour detection algorithm identifies connected components in *N* and traces the borders of these components. This process results in a set of coordinates that accurately define the boundary of each detected traffic sign $I_{d}$. By employing the contour detection algorithm, we can detect traffic signs within the binary masks, which is a key step in our TSD process. Then, we use the detected traffic sign $I_{d}$ to extract the traffic sign image *I* from real road images. In the following subsection, we introduce further details on the extraction of midlevel features from the detected traffic signs.

### 3.2. Midlevel Feature Extraction

**Figure 3:**
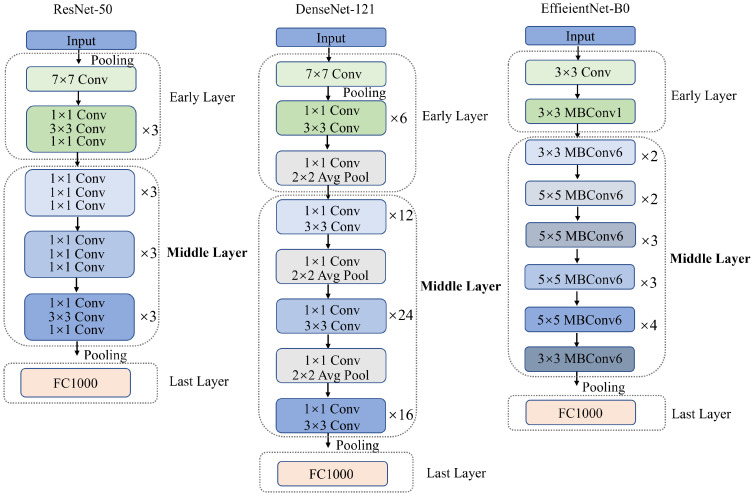
The structures of the early, midlevel, and last layers in different CNNs.

The extraction of midlevel features is crucial in our method. It employs zero-shot recognition by leveraging the inherent capabilities of pretrained CNNs, initially fine-tuned on the ImageNet dataset [59]. In this framework, we map traffic sign images onto different layers of these neural networks, covering a range from early layers, which capture basic features like shape, color, and edges to more advanced layers packed with semantic information [34,60]. The core of our method is to make use of the midlevel features inside CNNs. The middle layers effectively represent both high-level semantic meaning and basic characteristics such as shape and color, resulting in a complete and informative portrayal of traffic signs. To provide a clearer visual understanding of this process, Figure 3 illustrates the composition of our proposed CNN-based method. It shows the architecture of the early layers, midlevel layers, and final layers. The early layers, typically composed of a series of convolutional and pooling layers, are responsible for capturing the rudimentary characteristics of traffic signs [33,34]. These layers can capture basic visual features like edges, colors, and shapes.

In contrast, the midlevel layers play a significant role in capturing the rich semantic features of traffic signs. These layers contribute to the intricate fusion of shape, color, and semantic content, allowing the network to distinguish details that are crucial for traffic sign recognition. It is essential to mention that the number of layers and dimensions in these midlevel layers differs depending on different CNN architectures, such as ResNet-50, DenseNet-121, and EfficientNet-B0. This gives us the flexibility to choose a framework that works best for TSM.

The last layer typically comprises a fully connected layer, responsible for mapping the extracted midlevel features to specific traffic sign categories. This layer connects the neural network’s internal representations and the actual identification of traffic signs.

Table 1 details the dimensions of midlevel features within the proposed midlevel feature-based traffic sign recognition method. We show the dimensions of the optimal middle layer of different CNNs for traffic sign recognition. These dimensions reflect the richness and complexity of the information extracted by the network and show how the network transforms original road images into a structured and meaningful representation that facilitates accurate traffic sign recognition. The extraction of midlevel features is a fundamental aspect of the proposed method, enabling us to capture both low-level visual features and high-level semantic content from traffic sign images. This comprehensive representation forms the basis for our subsequent zero-shot matching process, which is at the core of the proposed TSM method.

The extraction of midlevel features *F* from a traffic sign image *I* using a CNN can be represented as follows:

(2) $$F_{\mathrm{mid}}\left(I\right)={\mathrm{CNN}}_{\mathrm{mid}}\left(I\right),$$

where ${\mathrm{CNN}}_{\mathrm{mid}}$ denotes the subnetwork capturing midlevel features. $F_{\mathrm{mid}}\left(I\right)$ represents the midlevel features of traffic signs by CNNs and is used for performing zero-shot matching.

### 3.3. Zero-Shot Matching

In this section, we delve into the zero-shot matching phase, which is an integral part of our traffic sign recognition approach based on midlevel feature representations. This recognition process can be divided into two main components: target traffic signs and template traffic signs. The former comprises traffic sign images from diverse geographical locales, while the latter represents an extensive repository of nationally sanctioned traffic sign templates.

The process begins by assessing the dissimilarity between the midlevel features of a target traffic sign $I_{\mathrm{target}}$ and those of the template traffic signs $T_{i}$, where *i* represents the index of the template traffic sign. This dissimilarity is calculated by the following Euclidean distance:

(3) $$\mathrm{Dissimilarity}\left(I_{\mathrm{target}},T_{i}\right)=\sqrt{\sum_{j=1}^{n}{\left(F_{\mathrm{mid}}\left(I_{\mathrm{target}}^{j}\right)-F_{\mathrm{mid}}\left(T_{i}^{j}\right)\right)}^{2}}.$$

Here, $F_{\mathrm{mid}}\left(I_{\mathrm{target}}\right)$ represents the midlevel features extracted from the target traffic sign, and $F_{\mathrm{mid}}\left(T_{i}\right)$ corresponds to the midlevel features of a template traffic sign $T_{i}$. This dissimilarity metric quantitatively measures how similar or dissimilar the features of the target sign are compared with the templates. We rank the top-*k* template traffic signs that exhibit the closest similarity to the target traffic sign. This ranking helps us identify the most likely matches among the template signs and provides a robust basis for recognizing the target sign.

Our novel approach to traffic sign recognition eliminates the traditional need for extensive training data. Instead, it leverages midlevel features to achieve recognition, making it adaptable to a wide range of traffic sign variations and locales. By focusing on feature similarity rather than explicit training on each sign type, our approach offers a versatile and effective solution to the challenges of traffic sign recognition.

## 4. Experiments

In this section, we show the experimental results and evaluations of our TSM method, demonstrating its effectiveness and robustness in real-world scenarios.

### 4.1. Experimental Settings

In this subsection, we provide an in-depth look at the experimental framework that forms the foundation of our study. Our experiments were conducted using the following two distinct datasets: the German Traffic Sign Recognition Benchmark (GTSRB) dataset [61] and a dataset consisting of urban road images from Sapporo City, Japan. These datasets were chosen to evaluate the effectiveness and robustness of the proposed TSM method. The GTSRB dataset comprises a diverse collection of 1,213 traffic sign images spanning 43 different classes. From this dataset, we handpicked 43 distinct classes of traffic signs as template traffic signs for the recognition task. The remaining images from the GTSRB dataset were designated as target traffic signs. It is noteworthy that the traffic sign images in the GTSRB dataset have already been extracted from road images, which aligns with our approach. For the Sapporo urban road dataset, we employed our ViT-Adapter-based method to meticulously extract traffic signs from the urban road images. This resulted in a final selection of 71 images representing 18 distinct types of traffic signs, all earmarked for use as target traffic signs in our experiments. Correspondingly, the template traffic signs for this dataset consisted of a comprehensive set of 111 categories, adhering to the prevailing traffic sign templates in Japan.

To assess the performance of the proposed TSM method, we conducted comparative analyses against conventional methods grounded in the HOG [11] and SIFT [3] techniques. This allowed us to benchmark our method against established approaches and highlight its advantages. For the extraction of midlevel features, we harnessed CNNs with ResNet-50, DenseNet-121, and EfficientNet-B0 architectures. It is crucial to note that all these networks were pretrained on the ImageNet dataset, and we left their inherent structures unaltered to ensure generality and applicability to various traffic sign recognition scenarios. To maintain uniformity and facilitate consistent analysis, we resized both target and template traffic sign images to the dimensions of 224 × 224 pixels. The evaluation metric we employed, Top-*k* accuracy, offers a comprehensive assessment of the method’s performance. This metric can be succinctly expressed as

(4) $$\mathrm{Top}-k=\frac{t_{k}}{\mathrm{Number}\;\mathrm{of}\;\mathrm{target}\;\mathrm{traffic}\;\mathrm{signs}}.$$

Here, $t_{k}$ represents the count of target images that successfully match templates within the Top-*k* matching results. Given the inherent challenges of zero-shot traffic sign recognition, including the absence of training data and the presence of highly similar template traffic signs, the Top-*k* metric serves as an effective gauge to measure the success of our zero-shot traffic sign recognition.

### 4.2. Experimental Results

In this section, we explore the experimental results and detailed analyses, shedding light on the effectiveness and adaptability of our TSM method across different datasets and scenarios.

The experimental results are shown in Table 2 and Table 3. In Table 2, we show the Top-*k* TSM results of different methods on the GTSRB dataset [61]. The proposed method based on the midlevel features of CNNs achieves promising results compared with the previous methods of using handcrafted features. The Top-*k* accuracy outperforms the comparative methods on three different CNNs, ResNet-50, DenseNet-121, and EfficientNet-B0, demonstrating the proposed method’s effectiveness. Furthermore, we also validated the recognition performance on the early layer, middle layer, and last layer of Table 1. The experimental results show that the proposed mid-level-feature-based method achieves the best TSM accuracy in all three CNNs. The results prove our hypothesis that the midlevel features can fuse the underlying information contained in the low-level features and the semantic information of the high-level features for better zero-shot traffic sign recognition.

To demonstrate the generality of the proposed traffic sign recognition method, we show the Top-*k* TSM results of different methods on the Sapporo urban road dataset in Table 3. Different from the experimental settings of the GTSRB dataset, the template traffic signs in the Sapporo urban road dataset are the common traffic sign templates in Japan. The Top-*k* accuracy of the proposed method for TSM in the Sapporo urban road dataset also outperforms the previous methods. In addition, the proposed mid-level-feature-based method also achieves the highest TSM accuracy on all three CNNs compared with the other layers, which further illustrates the effectiveness of the proposed method. It is worth mentioning that the recognition process does not require additional traffic sign images for training. The proposed method can obtain good feature representations of traffic sign images from different countries and achieve high accuracy for zero-shot traffic sign recognition.

**Figure 4:**
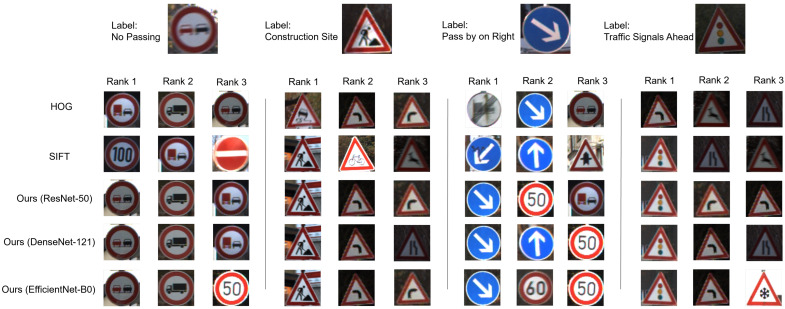
Examples of matching results for different methods on the GTSRB dataset.

Figure 4 presents illustrative matching results obtained with various methods on the GTSRB dataset. We show the matching results of four target traffic signs, deliberately chosen for their distinct colors and shapes, to underscore the efficacy of the proposed approach. The inclusion of the final target traffic sign, selected for its inherent blurriness, serves the purpose of evaluating the methods’ performance in handling ambiguous traffic signs.

As shown in Figure 4, the colors of the four target traffic signs in the GTSRB dataset encompass red, black, and blue, with shapes ranging from circular to triangular. The proposed mid-level-feature-based method consistently exhibits similar color and shape attributes in the top-matched template traffic signs across three neural networks, namely ResNet-50, DenseNet-121, and EfficientNet-B0. Furthermore, in comparison with manually crafted techniques such as HOG and SIFT, the proposed method also preserves semantic features. For instance, the central motif of the “No Passing” sign comprises vehicles, and the proposed method proficiently recognizes such semantic characteristics while retaining color and shape information. Notably, for the final example featuring a blurred traffic sign, the proposed CNN-based midlevel feature method accurately identifies the traffic sign, demonstrating its robustness. This characteristic is particularly pertinent in real-world scenarios, where signs may be subject to distortion or blurring due to adverse environmental conditions.

**Figure 5:**
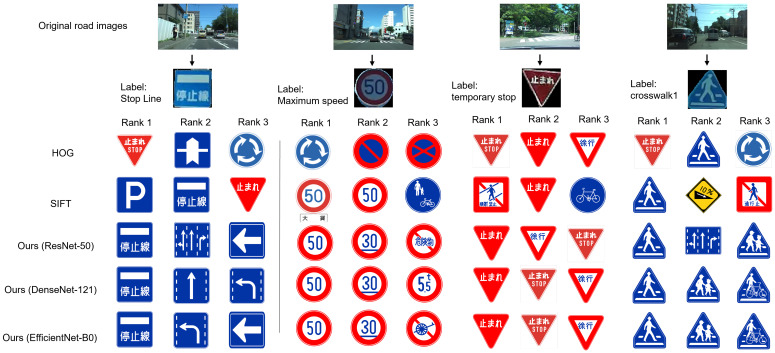
Examples of matching results for different methods on the Sapporo urban road dataset.

Figure 5 illustrates matching results obtained with different methods on the Sapporo urban road dataset. Given that this dataset comprises only original urban road images, we employed the ViT-Adapter and contour detection algorithms to extract traffic signs from the raw road images. We selected matching results for four target traffic signs to demonstrate the effectiveness of the proposed mid-level-feature-based TSM method. These target traffic signs exhibit various shapes, including rectangles, circles, and triangles, and come in different colors. As shown in Figure 5, the experimental results on the Sapporo urban road dataset reveal that the proposed midlevel feature method consistently exhibits similar color and shape attributes in the top-matched template traffic signs across three neural networks, namely ResNet-50, DenseNet-121, and EfficientNet-B0. Furthermore, in comparison with manually crafted techniques such as HOG and SIFT, the proposed method also preserves semantic features. For instance, the “Stop Line” and ”Temporary Stop” signs contain text, and the proposed method accurately recognizes this textual semantic feature while retaining color and shape information.

Additionally, the average computation time for traffic sign matching per target image in our proposed method, based on midlevel features for three different CNNs, is presented in Table 4. For the GTSRB dataset, the computation time per target image is 1.15 s for ResNet-50, 1.26 s for DenseNet-121, and 1.82 s for EfficientNet-B0. For the Sapporo urban road dataset, the computation time per target image increases to 3.63 s for ResNet-50, 4.01 s for DenseNet-121, and 5.35 s for EfficientNet-B0 due to the increased number of classes in the template traffic signs. Our approach exhibits reasonably efficient matching times across all three networks, which demonstrates the potential for application in traffic sign recognition within practical scenarios.

The proposed method consistently demonstrates similar matching results on examples from both datasets. This consistency underscores its effectiveness in traffic sign recognition across different scenarios. Specifically, on the GTSRB dataset, our method successfully matches target traffic signs with varying colors and shapes, showcasing its ability to handle diverse signage characteristics. It not only preserves color and shape information but also retains semantic features, making it a robust choice for real-world applications. Similarly, on the Sapporo urban road dataset, the proposed method exhibits remarkable performance in recognizing traffic signs with different shapes and colors, even when the signs are embedded within complex urban road scenes. Such versatility and reliability are essential for ensuring road safety and traffic management in urban environments. Our proposed mid-level-feature-based method consistently delivers robust matching results on both datasets, affirming its suitability for TSD and recognition tasks across diverse settings.

## 5. Discussion

In this section, we delve into a comprehensive discussion of the results obtained in our study, considering them in the context of prior research and our initial hypotheses. We also explore the broader implications of our findings and identify potential avenues for future research.

### 5.1. Interpretation of Results

Our study focused on the development and evaluation of a mid-level-feature-based method for traffic sign recognition using CNNs. The results presented in Table 2 and Table 3 and Figure 4 and Figure 5 demonstrate the robustness and effectiveness of our proposed method on two distinct datasets, GTSRB and the Sapporo urban road dataset. On the GTSRB dataset, our method consistently achieved accurate matching results for traffic signs with varying colors and shapes. This suggests that our approach can effectively handle the diversity of traffic signage encountered on real-world roads. Furthermore, our method can preserve both color and shape information, as well as semantic features, differentiating it from traditional handcrafted methods such as HOG and SIFT. Similarly, on the Sapporo urban road dataset, our method excelled in recognizing traffic signs within complex urban road scenes. The capacity to extract meaningful information from cluttered backgrounds is crucial for real-world traffic sign recognition systems, especially in urban environments.

### 5.2. Implications

The results of our study have several important implications. First, the proposed mid-level-feature-based method showcases the potential of leveraging CNNs for robust and versatile traffic sign recognition. This approach can be a valuable component of advanced driver assistance systems and autonomous vehicle technology, contributing to improved road safety. Second, our findings highlight the adaptability of our method to diverse datasets and scenarios. This adaptability is pivotal for real-world applications, where traffic signs can exhibit substantial variability in terms of appearance, lighting conditions, and environmental clutter.

### 5.3. Determining the Final Matched Traffic Signs

When determining the final matched traffic sign, it is important to consider the accuracy fluctuations within the Top-*k* rankings. The Top-*k* rankings are obtained based on the dissimilarity values between the target and each template traffic sign ($\mathrm{Dissimilarity}(I_{\mathrm{target}},T_{i})$). Lower dissimilarity values indicate higher rankings of the matched template traffic sign within the Top-*k* results. In practical applications in urban road scenarios, to assist drivers in making informed judgments in DASs, the final matched traffic sign from the Top-*k* matches can be guided by setting a dissimilarity threshold. When the dissimilarity value is below the threshold, the matched traffic signs are considered potential candidates for the final matched traffic sign. The proposed method is intended to assist the driver in making judgments. The threshold value varies based on actual road conditions, weather conditions, etc. For example, in clear weather conditions, where traffic signs are more easily recognizable, the threshold can be lower. In adverse road or weather conditions where signs may be blurry and harder to identify, the threshold can be higher.

### 5.4. Future Directions

Considering future directions, there are several avenues that warrant exploration. One particularly promising area is the integration of real-time video processing to extend the scope of our method for dynamic traffic sign recognition in video streams. Moreover, further refinement and optimization of the model architecture can enhance its efficiency and accuracy. Furthermore, enhancing the template database by incorporating a more diverse set of traffic sign variations and scenarios can significantly benefit the robustness and adaptability of our method. Rotating, distorting, or blurring the template traffic signs to simulate recognition under different road and weather conditions is also one of our future research directions. Meanwhile, we also need to consider the issue of traffic sign matching time, as an increase in the number of template traffic signs will escalate computation time, potentially compromising real-time performance. Additionally, incorporating more comprehensive and diverse datasets from different regions and countries can help validate the generalizability of our approach across various traffic sign standards and designs.

## 6. Conclusions

Our study presented a pioneering approach to zero-shot traffic sign recognition through a novel TSM method grounded in midlevel features. Through meticulous experimentation and analysis, we gained valuable insights into the capabilities of midlevel features extracted from CNNs. Our findings illuminate the significance of midlevel features, showcasing their proficiency in capturing both semantic and shape information intrinsic to traffic signs. This novel approach obviates the need for extensive training or fine-tuning on country-specific datasets, rendering it highly adaptable for traffic sign recognition across diverse geographical locales. In comparison with existing research, our work offers a fresh perspective on the challenges of traffic sign recognition. The TSM method, with its reliance on midlevel features, demonstrates superior adaptability and efficiency. This approach mitigates the need for extensive training, addressing a common limitation in current methods. This positions our study as a significant advancement, particularly in scenarios where access to large annotated datasets is constrained. The robustness and effectiveness of our method are underscored by the promising experimental results on two distinct datasets: the GTSRB and the Sapporo urban road dataset. These results demonstrate the method’s aptitude for accurate and efficient traffic sign recognition.

## Figures and Tables

**Table 1:** Dimensions of the different layers used in our method.

Network	Layer	Dimension
	Early layer	256 × 56 × 56
ResNet-50	Middle layer	1024 × 14 × 14
	Last layer	2048 × 1 × 1
	Early layer	256 × 56 × 56
DenseNet-121	Middle layer	1024 × 14 × 14
	Last layer	1024 × 7 × 7
	Early layer	16 × 112 × 112
EfficientNet-B0	Middle layer	320 × 7 × 7
	Last layer	1280 × 7 × 7

**Table 2:** Top-*k* accuracy of different methods on the GTSRB open dataset.

Method		Top1	Top5	Top10
HOG [11]		0.089	0.196	0.329
SIFT [3]		0.238	0.551	0.709
	Early Layer	0.141	0.352	0.569
ResNet-50	Middle Layer **(PM)**	**0.521**	**0.781**	**0.930**
	Last Layer	0.148	0.359	0.559
	Early Layer	0.081	0.227	0.374
DenseNet-121	Middle Layer **(PM)**	**0.468**	**0.769**	**0.910**
	Last Layer	0.394	0.680	0.864
	Early Layer	0.245	0.520	0.687
EfficientNet-B0	Middle Layer **(PM)**	**0.444**	**0.767**	**0.921**
	Last Layer	0.333	0.678	0.848

**Table 3:** Top-*k* accuracy of different methods on the Sapporo urban road dataset.

Method		Top1	Top5	Top10
HOG [11]		0.014	0.296	0.465
SIFT [3]		0.127	0.310	0.338
	Early Layer	0.014	0.211	0.338
ResNet-50	Middle Layer **(PM)**	**0.338**	**0.761**	**0.873**
	Last Layer	0.028	0.070	0.141
	Early Layer	0.014	0.141	0.268
DenseNet-121	Middle Layer **(PM)**	**0.817**	**0.873**	**0.915**
	Last Layer	0.169	0.606	0.732
	Early Layer	0.099	0.169	0.239
EfficientNet-B0	Middle Layer **(PM)**	**0.437**	**0.732**	**0.845**
	Last Layer	0.169	0.423	0.634

**Table 4:** Computation time per target traffic sign on two datasets across three different CNNs. “Class” represents the classes of template traffic signs.

Dataset	Class	Proposed Method	Computation Time (Seconds)
		ResNet-50	1.15
GTSRB	43	DenseNet-121	1.26
		EfficientNet-B0	1.82
		ResNet-50	3.63
Sapporo Urban Road	111	DenseNet-121	4.01
		EfficientNet-B0	5.35

## Data Availability

Publicly available datasets were analyzed in this study. These data can be found here: https://benchmark.ini.rub.de/ (accessed on 5 November 2013). Some data in this study were provided by Japan Radio Co., Ltd, Tokyo, Japan.

## Appendix A

**Table A1:** Abbreviations used in this article and their full forms.

Abbreviation	Full Form
CNNs	Convolutional Neural Networks
DASs	Driver Assistance Systems
SIFT	Scale-invariant Feature Transform
HOG	Histogram of Oriented Gradients
TSD	Traffic Sign Detection
TSC	Traffic Sign Classification
TSM	Traffic Sign Matching
GTSRB	German Traffic Sign Recognition Benchmark
